# American Veterinary Medical Association

**Published:** 1901-11

**Authors:** 


### AMERICAN VETERINARY MEDICAL ASSOCIATION.

### Resident State Secretaries for 1901.

Alabama—L. Van Es, Mobile.
Arizona—J. C. Norton, Phœnix.
Arkansas—R. R. Dinwiddie, Fayetteville.
British Columbia—Johnson Gibbins, 623 Granville Street, Vancouver.
California—Fred E. Pierce, 1724 Webster Street, Oakland.
Colorado—Charles Gresswell, 211 Whitney Building, Denver.
Connecticut—R. P. Lyman, 369 Allyn Street, Hartford.
Cuba and Porto Rico—C. D. McMurdo, Tenth United States Cavalry Manzanilla.
Delaware—H. P. Eves, 507 West Ninth Street, Wilmington.
District of Columbia—A. M. Farrington, 1436 Chapin Street, Washington.
Florida—J. G. Hill, 324 Forsythe Street, Jacksonville.
Georgia—N. P. Hinkley, Atlanta.
Hawaiian Islands—W. T. Monsarrat, Box 652, Honolulu.
Illinois—E. M. Nighbert, Mt. Sterling.
Indiana—J. R. Mitchell, Evansville.
Iowa—J. I. Gibson, Denison.
Kansas—N. S. Mayo, Manhattan.
Kentucky—J. W. Jameson, Paris.
Louisiana—E. Pegram Flower, 317 North Street, Baton Rouge.
Manitoba—W. J. Hinman, 277 James Avenue, Winnipeg.
Maryland—L. A. Nolan, corner of Dillon and Fifth Streets, Baltimore.
Massachusetts—Benjamin D. Pierce, 27 Sanford Street, Springfield.
Michigan—G. W. Dunphy, Quincy.
Minnesota—J. S. Butler, 40 South Seventh Street, Minneapolis.
Mississippi—J. C. Robert, Agricultural College.
Missouri—W. F. Heyde, 1215 South Jefferson Avenue, St. Louis.
Montana—Stewart W. McClure, Helena.
Nebraska—John D. Sprague, David City.
New Hampshire—Lemuel Pope, Jr., 101 State Street, Portsmouth.
New Jersey—J. Payne Lowe, 185 Jefferson Street, Passaic.
New York—William Henry Kelly, 233 Western Avenue, Albany.

North Carolina—A. S. Wheeler, Biltmore.
North Dakota—T. D. Hinebauch, Tower City.
Nova Scotia—William Jakeman, Halifax.
Ohio—F. E. Anderson, 514 South Main Street, Findlay.
Ontario—John W. Groves, 40 York Street, Hamilton.
Oregon—William McLean, 269 Market Street, Portland.
Pennsylvania—C. J. Marshall, 2004 Pine Street, Philadelphia.
Quebec—Charles H. Higgins, 6 Union Avenue, Montreal.
Rhode Island—Thomas E. Robinson, 65 Main Street, Westerly.
South Carolina—Benjamin McInnes, Charleston.
Tennessee—George R. White, 316 North Front Street, Nashville.
Texas—M. Francis, College Station.
Virginia—E. P. Niles, Blacksburg.
Washington—S. B. Nelson, Pullman.
West Virginia—L. N. Reefer, 1406 Chapline Street, Wheeling.
Wisconsin—R. H. Harrison, 83 Fourteenth Street, Milwaukee.

